# The relationship between personality traits and marital satisfaction: a systematic review and meta-analysis

**DOI:** 10.1186/s40359-020-0383-z

**Published:** 2020-02-07

**Authors:** Kourosh Sayehmiri, Karez Ibrahim Kareem, Kamel Abdi, Sahar Dalvand, Reza Ghanei Gheshlagh

**Affiliations:** 1 Health Faculty, Biostatistics Department, Ilam University of Medical Sciences, Ilam, Iran; 2 Department of Business Administration, College of Business and Economics, Bayan University, Erbil, Kurdistan Iraq; 3 Nursing Department, Komar University of Science and Technology, Sulimaniya, Iraq; 4 Department of epidemiology and Biostatistics, school of Public Health, Tehran University of Medical sciences, Tehran, Iran; 5 Social Determinants of Health Research Center, Research Institute for Health Development, Kurdistan University of Medical Sciences, Sanandaj, Iran

**Keywords:** Personality traits, Marital satisfaction, Meta-analysis, Iran

## Abstract

**Background:**

Personality traits can be used to predict an individual’s behaviors in different life situations, including marital life situations. Marital satisfaction that is influenced by different factors is a criterion used to assess couples’ relationship quality. The goal of the present study was to review Iranian studies on the correlation between personality traits and marital satisfaction.

**Methods:**

In this systematic review, all the related Iranian studies in international databases, including Google Scholar, PubMed, Web of Science (ISI) and Scopus, and national databases, including Scientific Information Database (SID) and MagIran were reviewed. The following keywords and also combinations of them were used to search the databases: “Marital satisfaction,” “Personality traits,” “Personality factors,” “Big five model of personality,” and “Iran.”

**Results:**

A total of 18 correlational studies, without any time limitation, with a total sample of 4049, were reviewed. The following correlation coefficients were found between marital satisfaction and personality traits: r = − 0.439 with neuroticism (95% Confidence Interval [CI]: 0.27–0.60), r = 0.833 with extraversion (95% CI: 0.77–0.88), r = 0.777 with openness (95% CI: 0.70–0.84), r = 0.855 with agreeableness (95% CI: 0.80–0.90), and r = 0.90 with conscientiousness (95% CI: 0.84–0.95).

**Conclusions:**

Couples high in Neuroticism experience lower levels of marital satisfaction, and couples high in Conscientiousness are more satisfied with their marital life.

## Background

Many couples see marriage as a sacred covenant that leads to family formation [1]. Stability of the family structure is dependent on the couple’s relationship quality. Dysfunctional marital relationships or unsuccessful marriages not only threaten couples’ mental health but also endanger the survival of the family unit [2]. Despite the fact that half of the first marriages in the United States end in divorce, the decision to divorce does not necessarily mean that people do not want to remarry and have a happy life. Therefore, two-thirds of couples in the US remarry within 5 years after divorce [3]. Marital satisfaction that results from sexual and emotional satisfaction is a measure of couples’ relationship quality, showing their subjective evaluation of the quality of their relationship [2, 4]. Marital satisfaction is a multidimensional concept comprising of different aspects of marital relationship, including adjustment, happiness, integrity, and commitment [5]. Marital satisfaction is a multidimensional concept comprising of different aspects of marital relationship [6].

Marital satisfaction is a mental state that is not achieved automatically, but requires the couple’s ongoing efforts to realize it, especially in the early years of marriage, because in this stage, marital satisfaction is unstable and marital relationship is at risk [7]. A couple experiences marital satisfaction when their marital relationship is consistent with what they had expected [2]. Marriage is a bond between two people with different personalities [8]. Claxton states that long-term and ideal romantic relationship requires that people, in assessment of their partner, go beyond physical characteristics, and consider personality traits [9]. Different factors, such as socioeconomic status, education, age, ethnicity, religious beliefs, physical attractiveness, Intelligence Quotient, and personal values and attitudes influence marital satisfaction, and can predict higher levels of marital satisfaction in couples [10].

Personality traits are among the factors influencing marital satisfaction. Karney et al. concluded that personality predicted life satisfaction [11]. Considering that people go into marriage with different personality traits, it can be said that marital relationship is a bond between two different personalities [12]. On the other hand, people tend to impose their behavioral and performance characteristics to their partner; therefore, their personality can act a stressor in their marital relationship [13]. For many years, it was a challenge for researchers to define personality, therefore different definitions were provided for this concept. Although there are many different personality traits, today, most researchers agree that the five-factor model can properly describe different aspects of personality [14]. According to this model, personality has five dimensions: neuroticism, extraversion, openness, agreeableness, and conscientiousness [15]. Neuroticism refers to one’s tendency to experience such feelings as anxiety, hostility, impulsivity, depression, and low self-esteem. Extraverts are more likely to be positive, assertive, and gregarious. Openness is related to such characteristics as curiosity, loving art, and wisdom. Agreeableness is related to such characteristics as kindness, generosity, empathy, and altruism. Conscientious people tend to be trustworthy and self-disciplined, and show aim for achievement People with different personality traits can have different attitudes toward different aspects of marital satisfaction [16].

In recent years, the number of marriages in Iran has declined for a variety of reasons, and divorce rates have increased significantly. Couples personality traits can be one of the factors associated with divorce and marital satisfaction. Iranian studies on this subject have reported inconsistent results. Some studies have not led to comprehensive results, and have only assessed the relationship of one personality trait (especially neuroticism) with marital satisfaction. Therefore, this systematic review and meta-analysis is aimed at examining the associations between marital satisfaction and personality traits in the Iranian population.

## Method

This is a systematic review and meta-analysis aimed at reviewing the relationship between personality traits and marital satisfaction reported by Iranian studies, without any time limitation, based on the Preferred Reporting Items for Systematic Reviews and Meta-Analyses (PRISMA) statement [17].

### Search strategy

International databases, including Google Scholar, PubMed, Web of Science (ISI) and Scopus, and national databases, including Scientific Information Database (SID) and MagIran were searched. The following keywords and also combinations of them were used to search the databases: “Marital satisfaction,” “Personality traits,” “Personality factors,” “Big five model of personality,” and “Iran.” In in the Persian databases, Persian equivalents of the keywords were used. Articles’ reference lists were also examined to find more related articles. Finally, a total of 18 articles were selected.

### Inclusion and exclusion criteria

We first collected all articles on the relationship between personality traits and marital satisfaction. Published in Persian and English and availability of the full text of the articles were among the inclusion criteria. Only the studies were included in the review that had used the NEO Five Factor Inventory (NEO-FFI) to assess personality traits and the ENRICH Marital Satisfaction Inventory to measure marital satisfaction. Studies that had not reported the correlation of all personality traits with marital satisfaction were excluded. Two authors of the present study independently reviewed the collected articles based on the inclusion and exclusion criteria. They first screened the articles based on titles and abstracts, and then reviewed the full text of the articles. Any disagreement between the two authors was resolved by asking the opinion of the third author.

### Data extraction

A form asking about the following information was used to extract data: Name of the first author, articles’ year of publication, articles’ country of publication, study design, and correlation coefficients reported between personality traits and marital satisfaction. The data were independently extracted by two authors, and when there was disagreement between them, were examined by the anothe author. In order to examine the methodological quality of the studies, an instrument commonly used in the Iranian and non-Iranian studies was used that assessed 5 aspects, including study design, comparison group, describing the characteristics of participants, sample size, and detailed description of the instruments used to gather data. Each aspect received a score from 0 to 3, and the total score ranged from 0 to 15. Based on methodological quality, the articles were divided into three categories: poor (0 to 5), average (6 to 10), and strong (above 10) [18, 19].

### Statistical analysis

The study data were analyzed using the random effects model (Mantel-Hansel). Using the normal distribution, the standard error of the mean was determined for each study. The effect size of each study was estimated using the following formula: $Z=0.5\ \mathrm{In}\frac{1+\mathrm{r}}{1-\mathrm{r}}$, where r is the correlation coefficient. The following formula was used to convert Z scores to r values: $r=\frac{\exp \left(2\mathrm{z}\right)-1}{\exp \left(2\mathrm{z}\right)+1}$. After converting the Z scores, the effect size pooled was obtained using the random effects model. The Chi-Square test with 0.1 significance level and the I^2^ index were used to assess heterogeneity among the studies. An I^2^ value of below 25% was considered as low heterogeneity, 25 to 75% as average heterogeneity, and above 75% as high heterogeneity [20]. The I^2^ index shows the proportion of observed differences among the reported indices that is due to heterogeneity among the studies. Heterogeneity was also examined using meta-regression and subgroup analysis. Univariate and multivariate methods were used to assess the causes of heterogeneity among the selected studies. In addition, the Egger’s test was used to examine potential publication bias.

## Results

**Fig. 1:**
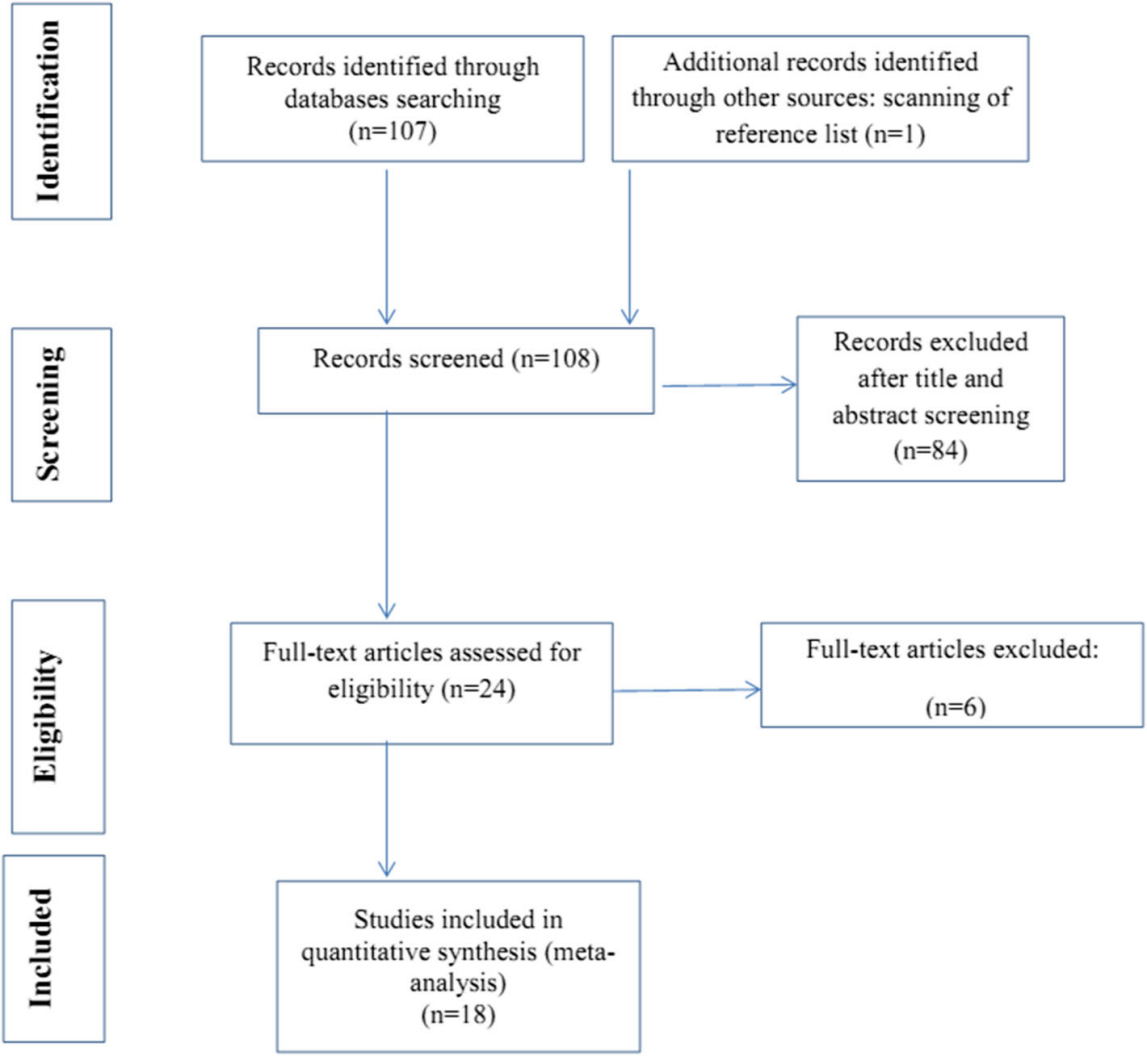
The screening process and selection of articles for meta-analysis based on the PRISMA guideline

In the initial search, 108 articles were identified, but 18 articles were selected using the inclusion criteria for final data analysis (Fig. 1).

Correlational studies without any time limitation were used. The total sample size was 4049 (mean: 225). The smallest (*n* = 43) and largest (*n* = 714) sample sizes were for the studies by Yaseminejad [21] and Sadeghi [22], respectively. Al the selected studies had average methodological quality. Table 1 shows the characteristics of the studies (Table 1).

**Table 1:** The characteristics of the selected articles for meta-analysis

First author	Year of publication	Sample size	Place of study	Language
Taragijah [23]	2017	200	Tabriz	Persian
Molaei [24]	2016	300	Tehran	English
Goodarzimehr [25]	2016	100	Babol	English
Sadeghi [22]	2016	714	Rasht	English
Etemadnia [26]	2015	153	Tonekabon	English
Khademi [1]	2015	400	Ahvaz	Persian
Ashoori [16]	2014	130	Tehran	Persian
Ghorbanzadeh [7]	2013	150	Ardabil	Persian
Javadmehr [27]	2013	70	–	English
Sadeghi [28]	2012	278	Rasht	English
Yasemini [21]	2011	43	–	Persian
Razeghi [29]	2011	200	Tehran	Persian
Amiri [30]	2011	100	Mashhad	Persian
Razavieh [31]	2011	112	Shiraz	Persian
Gholizadeh [12]	2010	160	Tabriz	Persian
Janati Jahromi [2]	2010	200	Kazerun	Persian
Ahadi [32]	2007	400	Tehran	Persian
Attari [33]	2006	339	Ahvaz	Persian

According to the results, the following correlation coefficients were found between marital satisfaction and personality traits: r = − 0.439 with neuroticism (with 95% Confidence Interval [CI]: 0.27–0.60), r = 0.833 with extraversion (with 95% CI: 0.77–0.88), r = 0.777 with openness (with 95% CI: 0.70–0.84), r = 0.855 with agreeableness (with 95% CI: 0.80–0.90), and r = 0.90 with Conscientiousness (with 95% CI: 0.84–0.95). Participants in the reviewed studies were divided into two populations: government employees and the general population. The findings based on these populations indicated that in both groups, the highest correlation was between marital satisfaction and conscientiousness, and the lowest correlation was between marital satisfaction and neuroticism. Findings based on article region indicated that in all regions of Iran (except for the region 2), the highest correlation was between marital satisfaction and conscientiousness, and the lowest correlation was between marital satisfaction and neuroticism. Findings based on article language showed that marital satisfaction had the highest correlation with conscientiousness and the lowest correlation with neuroticism. The Correlation between dimension of NEOs and Marital Satisfaction in all the Studied is presented in Table 2.

**Table 2:** The Correlation between dimension of NEOs and Marital Satisfaction in all the Studied Subgroups

Variable	Status	Dimension	No. of studies	Z-score	R	Confidence interval R(95%)		Heterogeniety	
						Lower	Upper	I^2^%	P
Population	Employee	Neuroticism	8	−0.17	0.500	0.161	0.819	97.1	< 0.0001
		Extraversion	8	0.23	0.882	0.803	0.947	79.1	< 0.0001
		Openness	8	0.09	0.777	0.639	0.876	84.6	< 0.0001
		Agreeableness	8	0.25	0.894	0.834	0.937	60.6	0.013
		Conscientiousness	8	0.27	0.906	0.833	0.946	55.9	0.026
	General	Neuroticism	10	−0.27	0.378	0.239	0.536	88.1	< 0.0001
		Extraversion	10	0.11	0.794	0.722	0.856	74.7	< 0.0001
		Openness	10	0.09	0.777	0.682	0.856	82.6	< 0.0001
		Agreeableness	10	0.15	0.826	0.759	0.888	76.6	< 0.0001
		Conscientiousness	10	0.26	0.900	0.810	0.963	89.6	< 0.0001
	Total	Neuroticism	18	−0.22	0.439	0.272	0.606	95	< 0.0001
		Extraversion	18	0.16	0.833	0.777	0.888	81	< 0.0001
		Openness	18	0.09	0.777	0.702	0.841	82.5	< 0.0001
		Agreeableness	18	0.19	0.855	0.802	0.900	77.1	< 0.0001
		Conscientiousness	18	0.26	0.900	0.848	0.942	83.4	< 0.0001
Region	Region 1	Neuroticism	7	−0.18	0.488	0.318	0.661	85.7	< 0.0001
		Extraversion	7	0.12	0.803	0.712	0.869	62.7	0.013
		Openness	7	−0.04	0.650	0.524	0.750	72	0.002
		Agreeableness	7	0.14	0.818	0.741	0.888	67.3	0.005
		Conscientiousness	7	0.22	0.875	0.794	0.932	72.1	0.001
	Region 2	Neuroticism	2	−0.35	0.284	0.058	0.595	81.1	0.021
		Extraversion	2	0.18	0.848	0.273	1.0391	95.3	< 0.0001
		Openness	2	0.33	0.937	0.712	1.029	85.8	0.008
		Agreeableness	2	0.19	0.856	0.712	0.951	55	0.136
		Conscientiousness	2	0.15	0.826	0.712	0.911	21.1	0.260
	Region 3	Neuroticism	5	−0.31	0.330	0.112	0.606	94.1	< 0.0001
		Extraversion	5	0.19	0.855	0.741	0.937	84.9	< 0.0001
		Openness	5	0.16	0.833	0.660	0.942	91	< 0.0001
		Agreeableness	5	0.22	0.875	0.759	0.951	85.9	< 0.0001
		Conscientiousness	5	0.35	0.946	0.833	1.01	92.2	< 0.0001
	Region 4	Neuroticism	2	0.08	0.768	0.01	1.051	99.3	< 0.0001
		Extraversion	2	0.31	0.927	0.888	0.964	0.0	0.429
		Openness	2	0.16	0.834	0.768	0.882	0.0	0.901
		Agreeableness	2	0.36	0.951	0.856	1.007	81.9	0.019
		Conscientiousness	2	0.40	0.967	0.937	0.991	0	0.717
	Unknown	Neuroticism	2	−0.37	0.261	0.272	0.606	0	0.574
		Extraversion	2	0.05	0.741	0.536	0.888	0	0.598
		Openness	2	0.13	0.810	0.628	0.927	0	0.518
		Agreeableness	2	0.14	0.818	0.639	0.937	0	0.918
		Conscientiousness	2	0.21	0.869	0.712	0.963	0	0.874
	Total	Neuroticism	18	−0.22	0.439	0.272	0.606	95	< 0.0001
		Extraversion	18	0.16	0.833	0.777	0.888	81	< 0.0001
		Openness	18	0.09	0.777	0.702	0.848	84.8	< 0.0001
		Agreeableness	18	0.2	0.862	0.810	0.906	79.5	< 0.0001
		Conscientiousness	18	0.28	0.911	0.855	0.951	86.6	< 0.0001
Language	Persian	Neuroticism	12	−0.22	0.439	0.198	0.682	96.4	< 0.0001
		Extraversion	12	0.21	0.869	0.794	0.927	81.4	< 0.0001
		Openness	12	0.13	0.811	0.702	0.894	88	< 0.0001
		Agreeableness	12	0.25	0.894	0.841	0.933	70.2	< 0.0001
		Conscientiousness	12	0.3	0.922	0.875	0.955	69.3	< 0.0001
	English	Neuroticism	6	−0.22	0.439	0.273	0.617	86.1	< 0.0001
		Extraversion	6	0.07	0.759	0.682	0.826	51.4	0.067
		Openness	6	0.03	0.722	0.617	0.811	68.1	0.008
		Agreeableness	6	0.1	0.786	0.682	0.876	76.5	0.001
		Conscientiousness	6	0.23	0.882	0.693	0.985	94.5	< 0.0001
	Total	Neuroticism	18	−0.22	0.439	0.272	0.606	95	< 0.0001
		Extraversion	18	0.16	0.833	0.777	0.888	81	< 0.0001
		Openness	18	0.09	0.777	0.702	0.848	84.8	< 0.0001
		Agreeableness	18	0.2	0.862	0.810	0.906	79.5	< 0.0001
		Conscientiousness	18	0.28	0.911	0.855	0.951	86.6	< 0.0001

## Discussion

The present study, aimed at examining the correlation between marital satisfaction and personality traits, indicated that marital satisfaction had a negative correlation with neuroticism; this finding is in line with the findings of a longitudinal study by Caughlin et al. [34]. In a longitudinal study by Fisher and McNulty with 72 couples in Ohio, United States, high levels of neuroticism predicted low levels of marital satisfaction 1 year later [35]. Individuals high in neuroticism often experience such feelings as sorrow, anger, and dissatisfaction with self, feelings that can reduce their overall happiness in life. Because these people are more likely to be moody and irritable, they are not expected to experience higher levels of marital satisfaction [36, 37]. Barelds showed that neuroticism reduced marital satisfaction [38]. In a study by Karney and Bradbury, neuroticism explained 10% of the variance of marital satisfaction [11]. Taraghijah notes that people high in Neuroticism feel less happiness because they put more emphasis on negative life events [23].

Other reasons for lower levels of marital satisfaction in people with high neuroticism include negative interpretation of ambiguous events [39], negative interactions with partner [40], negative interpersonal behavior during conflict and higher aggressive externalization [41], and lower levels of sexual satisfaction [35].

Attitude affects marital satisfaction. Due to the fact that attitude depends on personality, it can be concluded that people with different personality traits have different attitudes towards marriage and this can influence their marital satisfaction. In the present study, we aimed to examine the association between marital satisfaction and personality traits in Iranian older adults. People high in neuroticism tend to cope with life stressors less adaptively and are more likely to interpret normal situations as threatening or small frustrations as severe despair. Life satisfaction has many ups and downs that require patience and forgiveness from both partners, but people high in neuroticism tend to get stressed out and moody in the face of these problems, therefore jeopardizing not only their marital relationship but also their social and professional life.

The negative effects of neuroticism on marital satisfaction may be through creating anxiety, tension, pity-seeking, hostility, impulsivity, depression, and low self-esteem [32]. Personality traits like emotional instability and neuroticism may keep couples in a persistent state of vulnerability and influence the way they adapt to life stressors [42]. A 13-year longitudinal study among couples indicated that negative marital interaction resulted from high neuroticism. In other words, it was found that people high in neuroticism tend to display negative behavior towards their partners that in turn reduces marital satisfaction in both partners [34].

In the present study, a strong correlation was found between marital satisfaction and conscientiousness that is consistent with the findings of Claxton et al. [9] Given that conscientious people are self-disciplined, principled, and able to effectively handle relationship issues, they are expected to experience high levels of marital satisfaction [29]. Engel et al. (2002) found that conscientiousness was the best predictor of couple intimacy and commitment in men. They maintained that conscientious people show higher levels of intimacy in their relationships; therefore they are more able to build successful relationships [43]. In a study with 40–70-year-old couples who had been married more than 15 years, Shiota et al. (2007) found that marital satisfaction had the strong correlations with extraversion and conscientiousness [44]. In a longitudinal study conducted in Switzerland, satisfaction in intimate couples was positively correlated with agreeableness and conscientiousness [45]. Gattis et al. (2004) found that marital dissatisfaction was associated with low conscientiousness and high neuroticism [46]. People high in conscientiousness refrain from showing aggression and are capable of controlling their impulses in marital relationship [30]. Ahadi maintains that individuals low in conscientiousness resort to alcohol and physical aggression as a response to marital stressors, and this gradually reduces their marital satisfaction [32].

The present study was aimed at examining the relationship between marital satisfaction and personality traits in the Iranian population. The main limitation of this study was that most of the articles reviewed had not provided adequate information on the subject under study or had only examined the relationship of marital satisfaction with neuroticism, while ignoring the other four aspects. The other limitation was the lack of access to PsychInfo as a popular database. However, reviewing other articles and checking reference lists of the selected articles helped us make sure that the related studies conducted in Iran were collected and analyzed as much as possible.

## Conclusions

The study results indicated that couples high in neuroticism had low levels of marital satisfaction, while couples high in conscientiousness were more satisfied with their marital relationship. Because personality traits are relatively stable over time, they can be used to predict an individual’s behaviors in different life situations, including marital relationship. Examination of couples’ personality traits can improve our knowledge on the personality traits related to poor marital satisfaction that increase the risk of separation and divorce, and also the personality traits that are associated with a better, healthier, and more stable marital relationship.

Given the mutual relationship between personality and interpersonal relationships [47], healthcare providers, including psychologists and marriage counsellors need to pay more attention to these variables and the relationship between them, because evaluation of personality traits of both partners may have a considerable role in partner selection and also in avoiding marital maladjustment and marital satisfaction, and improper partner selection can lead to personality problems through creating marital maladjustment. The findings of the present study can help health care officials in analyzing personality traits and marital satisfaction, therefore, taking appropriate measures to strengthen the marital relationship of Iranian couples.

## Data Availability

The datasets used and/or analysed during the current study are available from the corresponding author on reasonable request.

## Abbreviations

CI: Confidence Interval
ISI: Web of Science
NEO-FFI: NEO Five Factor Inventory
PRISMA: Preferred Reporting Items for Systematic Reviews and Meta-Analyses
SID: Scientific Information Database
